# Safety of the BNT162b2 mRNA COVID-19 vaccine in oncologic patients undergoing numerous cancer treatment options: A retrospective single-center study

**DOI:** 10.1097/MD.0000000000028561

**Published:** 2022-01-14

**Authors:** Waleed Kian, Melanie Zemel, Emily H. Kestenbaum, Keren Rouvinov, Wafeek Alguayn, Dina Levitas, Anna Ievko, Regina Michlin, Moataz A. Abod, Ismaell Massalha, Elena Chernomordikov, Adam A. Sharb, Walid Shalata, Esther Levison, Laila C. Roisman, Konstantin Lavrenkov, Nir Peled, Lior Nesher, Alexander Yakobson

**Affiliations:** a The Legacy Heritage Oncology Center & Dr. Larry Norton Institute, Soroka Medical Center & Ben-Gurion University, Beer-Sheva, Israel; b Medical School for International Health, Ben-Gurion University of the Negev, Beer-Sheva, Israel; c Faculty of Health Sciences, Ben-Gurion University of the Negev, Beer-Sheva, Israel; d The Institute of Oncology, Shaare Zedek Medical Center, Jerusalem, Israel; e Infectious Disease Institute, Soroka Medical Center, Ben-Gurion University of the Negev, Beersheba, Israel.

**Keywords:** BNT162b2 mRNA, cancer, chemotherapy, COVID-19, immune checkpoint inhibitors, vaccine safety

## Abstract

The COVID-19 pandemic, caused by the SARS-CoV2 virus, has infected millions worldwide with cancer patients demonstrating a higher prevalence for severe disease and poorer outcomes. Recently, the BNT162b2 mRNA COVID-19 vaccine was released as the primary means to combat COVID-19. The currently reported incidence of local and systemic side effects was 27% in the general public. The safety of the BNT162b2 mRNA COVID-19 vaccine has not been studied in patients with an active cancer diagnosis who are either ongoing or plan to undergo oncologic therapy.

This single center study reviewed the charts of 210 patients with active cancer diagnoses that received both doses of the BNT162b2 mRNA COVID-19 vaccine. The development of side effects from the vaccine, hospitalizations or exacerbations from various oncologic treatment were documented. Type of oncologic treatment (immunotherapy, chemotherapy, hormonal, biologic, radiation or mixed) was documented to identify if side effects were related to treatment type. The time at which the vaccine was administered in relation to treatment onset (on long term therapy, within 1 month of therapy or prior to therapy) was also documented to identify any relationships.

Sixty five (31%) participants experienced side effects from the BNT162b2 mRNA COVID-19 vaccine, however most were mild to moderate. Treatment protocol was not linked to the development of vaccine related side effects (*P* = .202), nor was immunotherapy (*P* = .942). The timing of vaccine administered in relation to treatment onset was also not related to vaccine related side effects (*P* = .653). Six (2.9%) participants were hospitalized and 4 (2%) died.

The incidence of side effects in cancer patients is similar to what has been reported for the general public (31% vs 27%). Therefore, we believe that the BNT162b2 mRNA COVID-19 vaccine is safe in oncologic patients undergoing numerous cancer treatments.

## 1 Introduction

The COVID-19 pandemic caused by severe acute respiratory syndrome coronavirus 2 (SARS-CoV2) originated in Wuhan, China at the end of 2019. Since then, it has spread worldwide and infected hundreds of millions of people with millions of reported deaths.^[1]^ COVID-19 infects all age groups however there is a higher prevalence of severe disease in the elderly and those with comorbid conditions.^[2]^ Pregnancy, poor glycemic control, and cancer are associated with an increased susceptibility to acquiring COVID-19 and worse outcomes.^[3–5]^ Specifically, cancer patients have poorer outcomes and increased admittance to the intensive care unit (ICU) after contracting the disease compared with the general population (39% vs 8% respectively; *P* = .0003).^[6]^ In addition, cancer patients who underwent either surgery or chemotherapy within a month before infection had a significantly higher risk of severe adverse events than cancer patients who did not (OR 5.34, 95% CI 1.80–16.18, *P* = .0026).^[4]^

Currently, there are numerous cancer treatment options, including immune checkpoint inhibitors (ICIs), chemotherapy, target therapy and radiation therapy. ICIs are a leading form of cancer treatment that blocks several different proteins involved in cancer cells’ evasion of the immune system, allowing for the antitumor response to reactivate. These proteins include programmed cell death protein 1, programmed death ligand 1 and cytotoxic T-lymphocyte associated protein 4. ICIs are approved by the Food and Drug Administration for numerous types of malignancies such as non-small cell lung cancer, melanoma, breast cancer and others.^[7]^ Gambichler et al demonstrated that ICI therapy was not an independent risk factor for COVID-19 susceptibility in cancer patients.^[8]^ Another mainstay of treatment for most cancers includes chemotherapy. In addition to its toxic systemic side effects, patients undergoing active chemotherapy treatment displayed an increased risk of death from COVID-19 infection in a multicenter retrospective cohort study of 205 patients in China.^[9]^ Target therapy and radiation therapy are other common treatments used for various cancers, however they were not associated with increased severity of disease or fatality.^[10]^

The BNT162b2 mRNA COVID-19 vaccine is considered one of the most promising means for combating the COVID-19 pandemic. Towards the end of 2020, the BNT162b2 mRNA COVID-19 vaccines became available and distributed worldwide. The currently used two-dose mRNA-based vaccine encodes the coronavirus spike glycoprotein that mediates attachment to host cells. The safety of this vaccine has been studied and has been shown to produce mild to moderate side effects in more than half of the participants in a phase 1, dose-escalation, open-label trial that included 45 healthy adults. The most common of which were pain at the injection site, fatigue, headache, chills, and myalgia. These systemic side effects were even more common after the second dose of the vaccine; however, severe side effects were uncommon following either dose.^[11]^

Cancer patients are a crucial population to protect against the virus due to the increased risk of contracting COVID-19 and developing worse outcomes following the disease. However, to date, no studies have examined the safety of the BNT162b2 mRNA COVID-19 vaccine in oncologic patients, particularly ones that are undergoing treatment. Most cancer therapies alter the immune system's response to both pathogens and vaccines. This has been demonstrated when examining the immune response to the seasonal influenza vaccine. Chemotherapy has been shown to diminish the desired immunogenicity of the vaccine while ICI therapy, in itself, leads to a more robust immune response and has not been shown to alter the efficacy of the vaccine or lead to more severe side effects.^[12]^

With this in mind, we performed the following retrospective single center study to assess the safety of the BNT162b2 mRNA COVID-19 vaccine in oncologic patients that are undergoing treatment with ICIs, chemotherapy, targeted-therapy and/or radiation.

## 2 Methods

### 2.1 Study design

Retrospective single center study examining cancer patients from Soroka University Medical Center in Israel that assesses the safety of the BNT162b2 mRNA COVID-19 vaccine. Due to the lack of information about the vaccine safety, hospital protocol dictated close monitoring of vaccinated oncological patients by their treating physician. Physicians then documented any side effects, or any suspected treatment related adverse events following the BNT162b2 mRNA COVID-19 vaccine. This study reports on any side effects taking place after the administration of the first or second dose of the BNT162b2 mRNA COVID-19 vaccine. We also report if the BNT162b2 mRNA COVID-19 vaccine exacerbated any known side effects of numerus oncologic treatments. Side effects from the vaccine were graded based on a previously described scale.^[13]^ Adverse events from cancer therapy were graded according to the Common Terminology Criteria for Adverse Events. Pearson's Chi-Squared analysis was performed to identify differences in groups that reported or did not report any side effects. *P* values less than .05 were considered statistically significant. Statistics were done and the data was analyzed using IBM SPSS Statistics 26.0.

### 2.2 Ethics

This trial was conducted in accordance with the declaration of Helsinki and approved by Soroka University Medical Center ethics committee Sor-0016-21.

### 2.3 Participants

We collected data on patients with a confirmed malignancy that are currently being treated or planned to be treated at Soroka University Medical Center. Eligible patients were

1. 16 years or older with a confirmed malignancy,
2. actively being treated with chemotherapy, immune checkpoint inhibitors, target therapy or radiotherapy,
3. planned to be treated with chemotherapy, immune checkpoint inhibitors, target therapy or radiotherapy and
4. had received the BNT162b2 mRNA COVID-19 vaccine.

Patients were divided into the following groups: participants who received the vaccine (A) while on or (B) prior to antineoplastic therapy.

## 3 Results

We identified 210 patients who were vaccinated between December 20, 2020 (first date of vaccine availability) and April 30, 2021. A wide range of malignancies were included in this study with most solid malignancies being represented (Table 1).

**Table 1:** Demographics of participants in study. Participants that developed vaccine related side effects are also noted.

Characteristics	No. of patients (%) (N = 210)	No. Patients with vaccine side effects (%)^‡^ (N = 65)
Age		
Years	69 ± 11	65.2 ± 11
Sex		
Male	136 (64.8)	35 (25.7)
Female	74 (35.2)	30 (40.5)
Allergies		
Yes	47 (22.4)	14 (29.8)
No	163 (77.6)	51 (31.1)
Stage		
1	13 (6.2)	3 (23.1)
2	22 (10.5)	11 (50.0)
3	24 (11.4)	10 (41.7)
4	151 (71.9)	41 (27.2)
Treatment protocol		
Chemotherapy^∗^	42 (20.0)	16 (38.1)
Immunotherapy	48 (22.9)	12 (25.0)
Biological	24 (11.4)	9 (37.5)
Chemoimmunotherapy	20 (9.5)	9 (45.0)
Immuno-biological	9 (4.3)	4 (44.4)
Hormonal^∗^	43 (20.5)	7 (16.3)
Radiotherapy	3 (1.4)	2 (66.7)
Chemoradiotherapy	2 (1.0)	1 (50)
Immuno-radiotherapy	3 (1.4)	0 (0)
Chemo-biological	16 (7.6)	5 (31.3)
Radio-hormonal	1 (0.5)	0 (0)
Diagnosis^†^		
Lung cancer		
NSCLC	35 (16.6)	11 (31.4)
Small cell lung Ca	5 (2.4)	1 (20.0)
Skin cancer		
Melanoma	27 (12.9)	8 (29.6)
Basal cell Ca	6 (2.9)	1 (16.7)
Squamous cell Ca	3 (1.4)	0 (0)
Merkel cell Ca	1 (0.5)	0 (0)
Kaposi sarcoma	4 (1.9)	2 (50)
Epithelioid hemangioendothelioma	1 (0.5)	1 (100)
GU		
Breast	29 (13.8)	12 (41.4)
Ovarian	4 (1.9)	1 (25.0)
Endometrial	1 (0.5)	1 (100)
Fallopian tube	1 (0.5)	1 (100)
Prostate	43 (20.5)	7 (16.3)
Seminoma	1 (0.5)	1 (100)
Renal cell Ca	17 (8.1)	7 (41.2)
Transitional cell Ca	7 (3.3)	0 (0)
Gastrointestinal		
Esophageal	1 (0.5)	0 (0)
Gastric	10 (4.8)	2 (20.0)
GIST	1 (0.5)	0 (0)
Gastric signet ring	1 (0.5)	0 (0)
Colon Ca	12 (5.7)	6 (50.0)
Rectal	3 (1.4)	0 (0)
Hepatocellular Ca	1 (0.5)	1 (100)
Pancreatic Ca	2 (1.0)	1 (50.0)
Other		
Medullary thyroid	1 (0.5)	0 (0)
Parotid glad SCC	1 (0.5)	1 (100)
GBM	1 (0.5)	0 (0)
CLL	1 (0.5)	0 (0)
Follicular lymphoma	1 (0.5)	0 (0)

∗ 1 patient received chemotherapy and hormonal therapy for 2 different primaries.

† 9 patients had 2 primary cancers and 1 patient had 3 primary cancers.

‡ Percentage of patients with side effects by characteristic.

The average age of patients enrolled was 69 ± 11 with 136 (64.8%) participants being male and 74 (35.2%) participants being female. Of the 65 (31%) participants that experienced side effects, the average age was 65.2 ± 11, with 35 (25.7%) participants being male and 30 (40.5%) were female. Females reported experiencing significantly more side effects than males (*P* = .027, Table 3). Only 47 (22.4%) participants reported a history of allergic reactions, to either medication, food, or both, and of those 14 (29.8%) experienced a side effect following the vaccine. However, of the 163 (77.6%) participants that did not have a prior allergic reaction, 51 (31.1%) experienced a side effects to the vaccine.

Treatment protocols differed between participants and included chemotherapy, immunotherapy, biologics, hormonal therapy, and radiotherapy, along with combination therapy. Treatment protocol did not show to have an effect on the development of any side effects (*P* = .202). Forty two participants were on chemotherapy and 16 of those developed side effects. Forty eight participants were on immunotherapy and 24 were on biologic treatment, however only 12 and 9 of the participants on immunotherapy and biologic therapy, respectively, experienced any side effects. Forty three participants were on hormonal therapy and 3 were on radiotherapy and only 7 and 2 participants experienced side effects while on hormonal or radiotherapy, respectively. Of the combination treatment protocols, 20 patients received chemo-immunotherapy, 9 immuno-biological and 3 were on immuno-radiotherapy. Of those, 9, 4, and 0 participants experienced side effects, respectively. Participants in each treatment protocol can be seen in Table 1.

**Figure 1:**
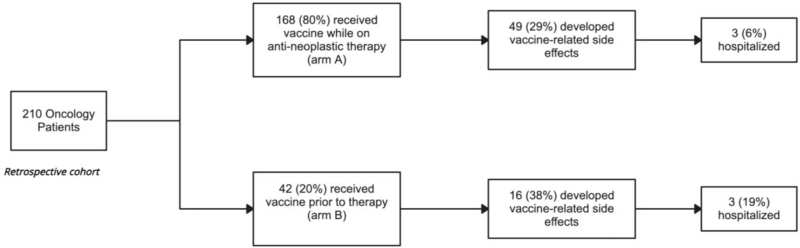
Patient allocation divided by time of vaccine administration in relation with initiation of systemic treatment.

One hundred sixty eight participants were on antineoplastic treatment at the time of vaccination (arm A) and 42 participants were vaccinated prior to the start of any therapy (arm B). For patients in arm A, 49 experienced any vaccine related side effects, of which, 30 and 31 patients demonstrated side effects following the 1st or 2nd vaccination, respectively (Fig. 1 and Table 2). In arm B, only 16 participants experienced any vaccine related side effects with 11 of these demonstrating side effects following either dose (Fig. 1, Table 2).

**Table 2:** Side effects based on timing of vaccination.

	All enrolled patients no. (%) (N = 210)	Enrolled patients with vaccine side effects no. (%) ^†^ (N = 65)
Received vaccine		
On long term systemic therapy (Ongoing)	152 (72.4)	45 (29.6)
Within 1 mo of therapy (New)	34 (16.2)	11 (32.4)
Prior to therapy (Prior)	24 (11.4)	9 (37.5)
Underwent radiation		
Within 1 mo of vaccine	25 (11.9)	10 (40.0)
≥1 mo after vaccine	12 (5.7)	5 (41.7)
Complications		
Therapy AE worsened	18 (8.6)	11 (61.1)
Therapy delay	10 (4.8)	9 (90.0)
Hospitalization	6 (2.9)	6 (100)
Death	4^∗^ (1.9)	4^∗^ (100)

Number of patients that experiences side effects after being split into ongoing, new and prior groups.

∗ 3 patients died from disease progression and 1 due to unknown causes.

† Percentage of patients with side effects by group.

However, there was no significant difference between when the participant received the vaccine and the onset of side effects (*P* = .653, Table 3). Of the 9 participants that received the vaccine prior to start of therapy, 7 of them were on a treatment protocol that included chemotherapy. Additionally, 18 participants experienced adverse events that were related to their oncologic therapy and of those 11 also experienced vaccine side effects (Table 2). Therapy delay occurred in 10 participants and 9 of those also experienced side effects from the vaccine. 6 participants were hospitalized during the study period and 4 participants died.

**Table 3:** Group differences in vaccine side effects.

	Number of participants	*P* value
Immunotherapy	80	.942
Non immunotherapy	130	
Vaccine on long term therapy	152	.653
Vaccine within 1 mo. of therapy	34	
Vaccine prior to therapy	24	
Males (with side effects)	136 (35)	.027
Females (with side effects)	74 (30)	

Chi-Squared analysis showed no difference in observed side effects when comparing immunotherapy treatment to nonimmunotherapy treatment or when comparing the time of administration of the vaccine in relation to initiation of oncologic therapy. Chi-Squared analysis demonstrated a significant difference between genders when reporting side effects.

65 (31%) participants experienced side effects from the vaccine. Of the side effects, most were grade 1 or grade 2 (n = 64) with the most prominent side effects described being pain at injection site (n = 30) after the first dose (Table 4). One patient experienced grade 3 to 4 fatigue following the first dose (Table 4). Following the second dose of the vaccine, the most participants again described grade 1 to 2 side effects (n = 61) with the most prominent side effect being injection site pain (n = 18) and fatigue (n = 18). 4 (1.9%) participants experienced grade 3 to 4 fatigue following the second dose (Table 4).

**Table 4:** Side effects following each dose of the BNT162b2 mRNA Covid-19 vaccine.

	Number of patients (%)			
	Dose 1		Dose 2	
Side effects of coronavirus vaccine (N = 65)	Grade 1–2	Grade 3–4	Grade 1–2	Grade 3–4
Injection site				
Pain	30 (46.2)		18 (27.7)	
Erythema	1 (1.5)		2 (3.1)	
Edema/Induration	1 (1.5)		2 (3.1)	
Pruritis	2 (3.1)		1 (1.5)	
Headache	2 (3.1)			
Myalgia	3 (4.6)		3 (4.6)	
Arthralgia	1 (1.5)		2 (3.1)	
Chills	2 (3.1)		9 (13.8)	
Diarrhea			1 (1.5)	
Fever	1 (1.5)		7 (10.8)	
Nausea				
Fatigue	12 (18.5)	1 (1.5)	18 (27.7)	4 (6.2)
Dysarthria	1 (1.5)			
Cough			2 (3.1)	
Sore throat			2 (3.1)	
Lymphadenopathy	1 (1.5)			
Weakness	1 (1.5)		1 (1.5)	
Allergic reaction				

## 4 Discussion

To the best of our knowledge, this is the first reported study to look at the safety of the BNT162b2 mRNA COVID-19 vaccine specifically within the cancer population. Both doses of the vaccine were generally well tolerated regardless of individual treatment protocol. The overall incidence of any side effects following either dose was 31.0% (n = 65). This value is similar to previously reported safety data which determined the incidence for side effects within the general population to be 27%, in a phase 3 trial enrolling 43,548 participants.^[13]^ Women tended to report side effects significantly more frequently than males (*P* = .027). This phenomenon has been reported following the H1N1 vaccine.^[14]^ Potential reasons include more frequent reporting or development of side effects following vaccines in women.^[15,16]^ A recently published prospective study of healthcare workers in South Korea showed no difference in frequency of side effects between genders with the BNT162b2 mRNA COVID-19 vaccine.^[17]^ However, their study included a vastly different population and the results cannot be generalized to oncologic patients.

The most reported local side effect was mild to moderate pain at the injection site (1st dose: n = 30; 2nd dose: n = 18). However, only 14% and 8.6% of participants reported pain at the injection site following either the first or second dose of the vaccine, respectively. These values are much lower than recently published side effect data in participants 55 years or older (71% following 1st dose, 66% following second dose).^[13]^ The most commonly reported systemic side effects included mild to moderate chills (1st dose: n = 2; 2nd dose: n = 9) and fatigue (1st dose: n = 12; 2nd dose: n = 18). Systemic side effects are more frequently reported following the second dose in the general population and this trend is also seen in our study.^[13]^

Treatment protocol did not show to have effect on developing side effects (*P* = .202, Table 3). Interestingly, patients on immunotherapy did not have more side effects than patients on all other treatments, even though an exaggerated immune response was predicted with this treatment (*P* = .942, Table 3).

At the data cutoff date, only 2 patients were diagnosed with covid-19 after administration of the vaccine. Neither of these patients reported any side effects to the vaccine and serum antibody levels to the vaccine were never checked. One patient was infected 8 days following only the 1st dose while the second patient was infected 2 days following the second dose. Both of these patients were not considered fully vaccinated as the peak antibody response is shown to be approximately 2 weeks after the second dose.^[18]^ These infections, despite immunization, raises the question of if cancer patients are able to develop a substantial immune response to the BNT162b2 mRNA COVID-19 vaccine. A recently published prospective study showed decreased efficacy of the vaccine in cancer patients actively undergoing treatment.^[19]^ Decreased immune response to vaccines in oncology patients has also already been demonstrated with different seasonal vaccinations.^[20–22]^ Even though these patients were not considered to be fully vaccinated, the efficacy of the vaccine in oncologic patients’ needs to be further evaluated.

Only 6 patients included in this study were hospitalized for exacerbation of known oncologic treatment related adverse events. These oncologic treatments related adverse events included, neutropenic fever, interstitial lung disease, pancytopenia and durvalumab induced immune thrombocytopenia. Three hospitalized patients received the vaccine prior to treatment, 1 received the vaccine within the same month and 1 was on long term therapy. The last hospitalized participant completed 2 years of immunotherapy for metastatic uveal melanoma 15 months prior to the vaccine. Since immunotherapy is known to have long lasting effects, it is plausible that the patient developed an exaggerated immune response to the vaccine due to the immunotherapy. Even though patients with allergies have been linked to having a higher rate of side effects following the vaccine, none of the hospitalized patients in our study had any known prior allergies.^[23]^ The length of hospitalization ranged from 3 days to 21 days. The hospitalized patients also experienced a delay in therapy that ranged from 7 days to a complete stop in treatment with many of them requiring a dose reduction as well.

4 (1.9%) participants died during the study period. Three of these participants died due to disease progression including, 2 with metastatic melanoma and 1 with metastatic prostate cancer. The last participant received the second dose of the vaccine 15 days prior to initiation of immuno-biologic (Atezolizumab and Bevacizumab) treatment for metastatic hepatocellular carcinoma. On the following day, the participant was admitted to the hospital with grade 3 weakness, abdominal pain, nausea, and vomiting. While hospitalized for 14 days, the patient's condition further deteriorated until they eventually died.

The main limitation of this study is the potential for recall and reporting bias due to the means of data collection. This could have resulted in overestimation or underestimation of the frequency and severity of side effects. Since at the time, little safety information was known, hospital protocol dictated this reporting method. To reduce recall bias, future studies could use a questioner or another standardized data collection method to encompass all currently known side effects from the vaccine. However, this bias may alter only the minor side effects as, all major events including admissions, delay of therapy or death were captured. Thus, our conclusion that the vaccine is safe is not changed by this bias. Another limitation is the unequal gender composition of the participants. Almost double the number of males were included in the study. However, females reported side effects significantly more frequently than males. The unequal sample size could skew this finding. Larger studies with equal groups that are designed to evaluate gender differences should be done to further comment on any differences that may exist. However, the vaccine was found to be safe in both genders and the sample size difference does not alter our conclusion.

## 5 Conclusions

Overall, this retrospective cohort study demonstrated that the BNT162b2 mRNA COVID-19 vaccine was safe in cancer patients. The side effects were similar to those previously reported within the general population. Although larger studies focusing on both efficacy and safety need to be completed, we believe that it is safe and advantageous for cancer patients to receive the vaccine.

## Author contributions

**Conceptualization:** Waleed Kian, Melanie Zemel, Keren Rouvinov, Moataz A Abod, Ismaell Massalha, Elena Chernomordikov, Adam A. Sharb, Walid Shalata, Laila C Roisman, Konstantin Lavrenkov, Nir Peled, Lior Nesher, Alexander Yakobson.

**Data curation:** Waleed Kian, Melanie Zemel, Emily H Kestenbaum, Keren Rouvinov, Dina Levitas, Anna Ievko, Regina Michlin, Moataz A Abod, Ismaell Massalha, Elena Chernomordikov, Adam A. Sharb, Walid Shalata, Laila C Roisman, Konstantin Lavrenkov, Nir Peled, Lior Nesher, Alexander Yakobson.

**Formal analysis:** Waleed Kian, Melanie Zemel, Emily H Kestenbaum, Keren Rouvinov, Wafeek Alguayn, Dina Levitas, Regina Michlin, Moataz A Abod, Ismaell Massalha, Elena Chernomordikov, Adam A. Sharb, Walid Shalata, Esther Levison, Laila C Roisman, Konstantin Lavrenkov, Nir Peled, Lior Nesher, Alexander Yakobson.

**Investigation:** Waleed Kian, Nir Peled, Alexander Yakobson.

**Methodology:** Waleed Kian, Nir Peled, Alexander Yakobson.

**Project administration:** Waleed Kian, Alexander Yakobson.

**Supervision:** Waleed Kian.

**Visualization:** Waleed Kian, Nir Peled, Lior Nesher, Alexander Yakobson.

**Writing – original draft:** Waleed Kian, Melanie Zemel, Emily H Kestenbaum, Keren Rouvinov, Wafeek Alguayn, Dina Levitas, Anna Ievko, Regina Michlin, Moataz A Abod, Ismaell Massalha, Elena Chernomordikov, Adam A. Sharb, Walid Shalata, Laila C Roisman, Konstantin Lavrenkov, Nir Peled, Lior Nesher, Alexander Yakobson.

**Writing – review & editing:** Waleed Kian, Melanie Zemel, Wafeek Alguayn, Dina Levitas, Anna Ievko, Regina Michlin, Moataz A Abod, Ismaell Massalha, Elena Chernomordikov, Adam A. Sharb, Walid Shalata, Esther Levison, Laila C Roisman, Konstantin Lavrenkov, Nir Peled, Lior Nesher, Alexander Yakobson.
